# Supplementary material for: Inactivation of the Fusobacterium nucleatum Rnf complex reduces FadA-mediated amyloid formation and tumor development
Source: mBio. 2025 May 22;16(6):e01032-25. doi: 10.1128/mbio.01032-25 (PMC12153266; doi:10.1128/mbio.01032-25)
Supplement: Supplemental tables — Tables S1 to S3. [file mbio.01032-25-s0001.pdf]

Inactivation of the *Fusobacterium nucleatum* Rnf complex reduces FadA-mediated amyloid formation and tumor development

Timmie A. Britton[1], Ju Huck Lee[2,3], Chungyu Chang[4], Aadil H. Bhat[4], Yi-Wei Chen[4], Rusul Mohammed Ali[5], Chenggang Wu[2], Asis Das[6], and Hung Ton-That[1,4,5]

[1]*Molecular Biology Institute, University of California, Los Angeles, California, USA;* [2]*Department of Microbiology & Molecular Genetics, University of Texas McGovern Medical School, Houston, Texas, USA;* [3]*Korean Collection for Type Cultures, Korea Research Institute of Bioscience and Biotechnology, Daejeon, Republic of Korea*; [4]*Division of Oral & Systemic Health Sciences, School of Dentistry, University of California, Los Angeles, California, USA;* [5]*Department of Microbiology, Immunology & Molecular Genetics, University of California, Los Angeles, Los Angeles, CA, USA;* [6]*Department of Medicine, Neag Comprehensive Cancer Center, University of Connecticut School of Medicine, Farmington, CT, USA*

[1]To whom correspondence may be addressed; email htonthat@dentistry.ucla.edu

Running Title: Rnf-associated amyloid and tumor formation

Keywords: Rnf complex, metabolism, two-component system, amyloid, tumor, colorectal cancer

Supporting Tables

Table S1: Response Regulators in *F. nucleatum* ATCC 23726

Gene ID	Homolog Type	Associated Process[a]	Reference
C4N14_RS05840	ArlR	Adhesion, autolysis, proteolysis	(1, 2)
C4N14_RS09320	CarR	Lysine metabolism, coaggregation	(3)
C4N14_RS07475	CheY	Chemotaxis	(4)
C4N14_RS02760	EutV	Ethanolamine utilization	(5)
C4N14_RS04330	ModR	Oxidative stress response	(6)
C4N14_RS02660	S1 RNA binding	Transcription	This study
C4N14_RS04495	YpdB	Autolysis	(7)

[a]Based on the available information of homologous response regulators.

Table S2: Bacterial strains and plasmids used in this study

Strains & Plasmids	Description	Reference
Strain		
F. nucleatum ATCC 23726	Wild-type (WT) strain	(8)
F. nucleatum CW1	Δ*galK*; an isogenic derivative of 23726	(8)
F. nucleatum fap2::Tn5	Derivative of 23726 with Tn5 insertion mapped to *fap2* at position 2054 (11361)	(3)
F. nucleatum Δ*rnfC*	Isogenic derivative of CW1 lacking *rnfC*	(9)
F. nucleatum Δ*fadA*	Isogenic derivative of CW1 lacking *fadA*	This study
F. nucleatum Δ*carR*	Isogenic derivative of CW1 lacking *carR*	(3)
F. nucleatum Δ*modR*	Isogenic derivative of CW1 lacking *modR*	(6)
F. nucleatum Δ*arlR*	Isogenic derivative of CW1 lacking *arlR*	This study
F. nucleatum Δ*cheY*	Isogenic derivative of CW1 lacking *cheY*	This study
F. nucleatum Δ*eutV*	Isogenic derivative of CW1 lacking *eutV*	This study
F. nucleatum Δ*s1*	Isogenic derivative of CW1 lacking *s1*	This study
F. nucleatum Δ*ypdB*	Isogenic derivative of CW1 lacking *ypdB*	This study
Plasmid		
pCWU6	Derivative of pHS30	(8)
pMCSG7	ligation-independent cloning vector	(10)
pZP4C	CRISPR-based vector	(11)
pCM-GalK	*C. perfringens* vector expressing *galK*	(8)
pMCSG7-FadA	pMCSG7 expressing FadA	This study
pMCSG7-CarR	pMCSG7 expressing CarR	This study
pMCSG7-Fap2	pMCSG7 expressing Fap2	This study
pRnfC	pCWU6 expressing RnfC under the control of the *rpsJ* promoter	(9)
pZP4C-lepB	Derivative of pZP4C for *lepB* depletion	This study
pGalK-Δ*fadA*	pCM-galK derivative; *rnfC* deletion vector	This study
pGalK-Δ*arlR*	pCM-galK derivative; *arlR* deletion vector	This study
pGalK-Δ*cheY*	pCM-galK derivative; *cheY* deletion vector	This study
pGalK-Δ*eutV*	pCM-galK derivative; *eutV* deletion vector	This study
pGalK-Δs*1*	pCM-galK derivative; *s1* deletion vector	This study
pGalK-Δ*ypdB*	pCM-galK derivative; *ypdB* deletion vector	This study

Table S3: Primers used in this study

Primer	Sequence	Used for
rnfC-up-F	GGCGGGATCCATGAACTTTGAAGAAATAGATTTTTATATT	pGalK-Δ*rnfC*
rnfC-up-R	GGCGGGATCCTTAAAGGAGCTCCTATATGTTGTAAAAG	pGalK-Δ*rnfC*
rnfC-dn-F	GGCGGGTACCGTCCTATGGGGCTTGCACCACTTATG	pGalK-Δ*rnfC*
rnfC-dn-R	GGCGAAGCTTGCTAGTTGCTTCTGGTAAAACTTCTTTT	pGalK-Δ*rnfC*
Com-rnfC-F	GGCGGGTACCGGATAGTAGAAGTGCATTTAAAGATT	pRnfC
Com-rnfC-R	GGCGGGATCCCTACTTTTTCTTAGCTCTTAATTTAG	pRnfC
fadA-up-F	GGCGGGTACCCTTAACATAAGTTTACTATGATTT	pGalK-Δ*fadA*
fadA-up-R	TAATAGCTTTTTGTTGTTCCGAAGCAGAAACAGCTAATACTGC	pGalK-Δ*fadA*
fadA-dn-F	GGAACAACAAAAAGCTATTATTTC	pGalK-Δ*fadA*
fadA-dn-R	CGCGGATCCCTTCTTCTTCAGCATATTCAATTC	pGalK-Δ*fadA*
LIC-FadA-F	TACTTCCAATCCAATGCAATGCAAAAGGAAAAAGAACTTTC	pMCSG7-FadA
LIC-FadA-R	TTATCCACTTCCAATGTTACTATCTTATTTTTTGAATTTTTTC	pMCSG7-FadA
LIC-Fap2-F	TACTTCCAATCCAATGCAGATAATTATGGACAAATAACAGGTGTTGCAATG	pMCSG7-FadA
LIC-Fap2-R	TTATCCACTTCCAATGTTATTAGAATATAACTCTTAGTCCTACTCCACCTC	pMCSG7-FadA
LIC-CarR-F	TACTTCCAATCCAATGCA AAAATTTTAGTAGTTGAAGATG	pMCSG7-CarR
LIC-CarR-R	TTATCCACTTCCAATGTTATTCATCTTCTTTAAGAACATAGCC	pMCSG7-CarR
arlR-up-F	GGCGGGTACCCTGTATAGAATATAAAGCTATGGA	pGalK-Δ*arlR*
arlR-up-R	CTCTTGAACAAGACAACCCCAAATTTTATTCATCTTCCTCACCC	pGalK-Δ*arlR*
arlR-dn-F	GGGGTTGTCTTGTTCAAGAGA	pGalK-Δ*arlR*
arlR-dn-R	CGCGGATCCCAAAACTTAAATTATCTATAACTTC	pGalK-Δ*arlR*
cheY-up-F	CGCGGATCCCAAAACTTAAATTATCTATAACTTC	pGalK-Δ*cheY*
cheY-up-R	CATAAATACTCATTTCTGGGTCTACCTTCCTCCATTGAAAGGGC	pGalK-Δ*cheY*
cheY-dn-F	GTAGACCCAGAAATGAGTATTTATG	pGalK-Δ*cheY*
cheY-dn-R	AGAGTCGTCGACCAATGATTGCAATAGTTACAGGTG	pGalK-Δ*cheY*
eutV-up-F	AGAGGAGCTCGATTTACCTTTTGATGAAAGAG	pGalK-Δ*eutV*
eutV-up-R	AGAGGGTACCCTTGTAAGTGTTTCATCTTCCAC	pGalK-Δ*eutV*
eutV-dn-F	AGAGGGTACCGAAAGAGCCAAAGGAATAGTTATG	pGalK-Δ*eutV*
eutV-dn-R	AGAGGTCGACCTAAGTAAACTTGCCACTGTCTG	pGalK-Δ*eutV*
s1-up-F	AGAGGAGCTCCAGAAGATGCTACAACTGATG	pGalK-Δ*s1*
s1-up-R	AGAGGGTACCCTGTACTTCATCTAAATTTCCTG	pGalK-Δ*s1*
s1-dn-F	AGAGGGTACCGGAAGTAGGAATGGAACTTG	pGalK-Δ*s1*
s1-dn-R	AGAGGTCGACCTAGGTAATAGTAATCATCACC	pGalK-Δ*s1*
ypdB-up-F	GGCGGAGCTCGTGAAATTGTAGCAGGAAACCAAG	pGalK-Δ*ypdB*
ypdB-up-R	GGCGGGTACCCTCTTGCAGGCAATTCATCTTC	pGalK-Δ*ypdB*
ypdB-dn-F	GGCGGGTACCCTATGCAACAGCTATACCTGTAAG	pGalK-Δ*ypdB*
ypdB-dn-R	GGCGGTCGACCTTAGGCAGTTCAAGAGAGG	pGalK-Δ*ypdB*
RT-fadA-F	CAAGCTGACGCTGCTAGA	RT-PCR *fadA*

RT-fadA-R	GCTTGAAGTCTTTGAGCTCTTT	RT-PCR *fadA*
RT-carR-F	TTGACAGCAAGAGATGGAATAG	RT-PCR *carR*
RT-carR-R	CTTCTCACAAGAGCTCTAATTCT	RT-PCR *carR*
RT-modR-F	CTTCAACGGGAGAAGAGGCA	RT-PCR *modR*
RT-modR-R	AGCCGTTTGTGCATAATCAAAAT	RT-PCR *modR*
RT-arlR-F	CTTGGGTGAGGAAGATGAATAA	RT-PCR *arlR*
RT-arlR-R	CATAAGCAGAGTCAACAGAGTAG	RT-PCR *arlR*
RT-cheY-F	ATGGTGTGTTGGGAGTAAAG	RT-PCR *cheY*
RT-cheY-R	TGCTCCTGCTTCAAAGAAA	RT-PCR *cheY*
RT-eutV-F	GGCTATGATGTTGTAGGAGAAG	RT-PCR *eutV*
RT-eutV-R	TTAGCAACCTTTAGTCCAGAAA	RT-PCR *eutV*
RT-s1-F	GGAACGGCTTCAAGAGAAA	RT-PCR *s1*
RT-s1-R	AGCTCCTGCTTCATTAACTATC	RT-PCR *s1*
RT-ypdB-F	GCCTGATATGAATGGAATTAGTC	RT-PCR *ypdB*
RT-ypdB-R	TTTATCTCAAAGGCATCAACAG	RT-PCR *ypdB*
RT-radD-F	GCAGCAGCACCAACAATAAAT	RT-PCR *radD*
RT-radD-R	GGTGCTTCAGGAGGTGTTATC	RT-PCR *radD*
RT-16s-F	GGTTAAGTCCCGCAACGA	RT-PCR *16s*
RT-16s-R	CATCCCCACCTTCCTCCTAC	RT-PCR *16s*
Sg-lepB_F	CCAAATTTCCTATATAAAATCTTGTTTTAGAGCTAGAAAT AGCAAGTT	CRISPRi-lepB
Sg-RNA-R	GATCCGCGGCCGCTAGTCAG	CRISPRi-lepB

[a] Underlined are restriction site sequences.

References

1. Fournier B, Hooper DC. 2000. A new two-component regulatory system involved in adhesion, autolysis, and extracellular proteolytic activity of *Staphylococcus aureus*. J Bacteriol 182:3955-64. https://doi.org/10.1128/JB.182.14.3955-3964.2000.
2. Fan R, Li Z, Shi X, Wang L, Zhang X, Dong Y, Quan C. 2022. Expression, Purification, and Characterization of the Recombinant, Two-Component, Response Regulator ArlR from *Fusobacterium nucleatum*. Appl Biochem Biotechnol 194:2093-2107. https://doi.org/10.1007/s12010-021-03785-5.
3. Wu C, Chen YW, Scheible M, Chang C, Wittchen M, Lee JH, Luong TT, Tiner BL, Tauch A, Das A, Ton-That H. 2021. Genetic and molecular determinants of polymicrobial interactions in *Fusobacterium nucleatum*. Proc Natl Acad Sci U S A 118. https://doi.org/10.1073/pnas.2006482118.
4. Stock A, Koshland DE, Jr., Stock J. 1985. Homologies between the *Salmonella typhimurium* CheY protein and proteins involved in the regulation of chemotaxis, membrane protein synthesis, and sporulation. Proc Natl Acad Sci U S A 82:7989-93. https://doi.org/10.1073/pnas.82.23.7989.
5. Fox KA, Ramesh A, Stearns JE, Bourgogne A, Reyes-Jara A, Winkler WC, Garsin DA. 2009. Multiple posttranscriptional regulatory mechanisms partner to control ethanolamine utilization in *Enterococcus faecalis*. Proc Natl Acad Sci U S A 106:4435-40. https://doi.org/10.1073/pnas.0812194106.
6. Scheible M, Nguyen CT, Luong TT, Lee JH, Chen YW, Chang C, Wittchen M, Camacho MI, Tiner BL, Wu C, Tauch A, Das A, Ton-That H. 2022. The Fused Methionine Sulfoxide Reductase MsrAB Promotes Oxidative Stress Defense and Bacterial Virulence in *Fusobacterium nucleatum*. mBio 13:e0302221. https://doi.org/10.1128/mbio.03022-21.

7. Nikolskaya AN, Galperin MY. 2002. A novel type of conserved DNA-binding domain in the transcriptional regulators of the AlgR/AgrA/LytR family. Nucleic Acids Res 30:2453-9. https://doi.org/10.1093/nar/30.11.2453.
8. Wu C, Al Mamun AAM, Luong TT, Hu B, Gu J, Lee JH, D'Amore M, Das A, Ton-That H. 2018. Forward Genetic Dissection of Biofilm Development by *Fusobacterium nucleatum*: Novel Functions of Cell Division Proteins FtsX and EnvC. mBio 9. https://doi.org/10.1128/mBio.00360-18.
9. Britton TA, Wu C, Chen YW, Franklin D, Chen Y, Camacho MI, Luong TT, Das A, Ton-That H. 2024. The respiratory enzyme complex Rnf is vital for metabolic adaptation and virulence in *Fusobacterium nucleatum*. mBio 15:e0175123. https://doi.org/10.1128/mbio.01751-23.
10. Siegel SD, Amer BR, Wu C, Sawaya MR, Gosschalk JE, Clubb RT, Ton-That H. 2019. Structure and Mechanism of LcpA, a Phosphotransferase That Mediates Glycosylation of a Gram-Positive Bacterial Cell Wall-Anchored Protein. mBio 10. https://doi.org/10.1128/mBio.01580-18.
11. Zhou P, G CB, Stolte F, Wu C. 2024. Use of CRISPR interference for efficient and rapid gene inactivation in *Fusobacterium nucleatum*. Appl Environ Microbiol 90:e0166523. https://doi.org/10.1128/aem.01665-23.
